# Risk Factors for Ovarian Cancer in South America: A Literature Review

**DOI:** 10.3390/jpm14090992

**Published:** 2024-09-18

**Authors:** Sergio Jara-Rosales, Roxana González-Stegmaier, Elena S. Rotarou, Franz Villarroel-Espíndola

**Affiliations:** 1 Faculty of Health Care Sciences, School of Midwifery, Universidad San Sebastián, Los Leones Campus, Santiago 7510157, Chile; sergio.jara@uss.cl; 2 Doctorate Program in Chronic Diseases, Faculty of Medicine and Science, Universidad San Sebastián, Los Leones Campus, Santiago 7510157, Chile; elena.rotarou@uss.cl; 3 Translational Medicine Laboratory, Instituto Oncológico Fundación Arturo López Pérez, Santiago 7500921, Chile; roxana.gonzalez@falp.org; 4 Faculty of Medicine and Science, Universidad San Sebastián, Los Leones Campus, Santiago 7510157, Chile

**Keywords:** ovarian cancer, risk factors, *BRCA1* and *BRCA2*, obesity, South America

## Abstract

**Background/Objectives:** In 2020, ovarian cancer ranked fourth in global incidence among gynecological cancers and remains the deadliest cancer affecting women’s health. Survival rates are significantly higher when the disease is detected at early stages; however, the lack of effective early detection methods underscores the importance of identifying risk factors in order to implement preventive strategies. The objective of this work is to provide an overview of the risk factors of ovarian cancer in South America, emphasizing those linked to social determinants, genetic components, and comorbidities. **Methods:** A literature search was performed using PubMed and Google Scholar. MeSH descriptors and keywords, such as “*BRCA1* genes,” “*BRCA2* genes”, “Latin America”, and “ovarian neoplasms” were used, along with terms related to socioeconomic and health factors. Inclusion criteria focused on original studies published in the last five years involving South American women. **Results:** Studies were identified from Argentina, Brazil, Chile, Colombia, Ecuador, and Peru. These studies addressed genetic factors, health status at diagnosis, and sociodemographic factors, revealing important data gaps, particularly on contraception and hormone replacement therapy. The prevalence of *BRCA1* and *BRCA2* mutations in South America is estimated to be 15–20% among women with inherited risk factors. Social, demographic and economic factors vary by country, although commonalities include a higher prevalence among women over 50 years of age, those with limited education, and those who face barriers to accessing health care. **Conclusions:** Although the literature does not conclusively establish a direct link between obesity and/or diabetes and the development of ovarian cancer, the indirect association highlights the need for further clinical studies. A general research gap related to risk factors of ovarian cancer could be observed in the South American region.

## 1. Introduction

In 2020, ovarian cancer ranked fourth in incidence among gynecological cancers worldwide, following breast cancer, cervical cancer, and uterine cancer [1]. In terms of mortality, it ranked third, establishing itself as one of the most lethal gynecological cancers [1]. A 2021 study revealed that the variability in the incidence depends on each country and region, but the mortality rate remains similar among the 185 countries included [1]. This behavior is attributed to nonspecific symptoms and the lack of screening programs, leading to diagnoses at advanced stages with unfavorable prognoses for patients [2,3].

Ovarian cancer constitutes a group of diverse diseases, each characterized by its own morphology and biological behavior. This heterogeneity implies that different subtypes may respond differently to treatments and show variations in their progression rates [4]. It is therefore essential to recognize this diversity to effectively address the different manifestations of ovarian cancer.

The risk factors for ovarian cancer are divided into two categories: modifiable and non-modifiable. Modifiable factors include smoking, hormone replacement therapy, and dietary elements, among others. Non-modifiable factors include heredity, mutations in the *BRCA1* and *BRCA2* genes, family history, Lynch syndrome, uninterrupted ovulation cycles, and the presence of endometriosis, among others [3,5].

The Latin American and Caribbean population constitutes a large heterogeneous group with diversity in national origin, racial makeup, genetic ancestry, modifiable cancer risk factors, environmental exposures, socioeconomic status, and acculturation, that share both cultural similarities and differences. Although the terms Hispanic and Latin are often used interchangeably, they have different meanings and histories and refer to different ancestral origins. Hispanic refers to people with Spanish heritage, while Latin refers to individuals related by the Latin language, including non-Spanish-speaking groups, such as Haitians and Brazilians. In fact, Latin America can be segregated into three parts: the Caribbean, Central America, and South America, increasing the variability and complexity of studying the cancer biology of these human groups.

The risk factors present differences according to the geographical regions analyzed, and so far, information is limited regarding how reproductive and lifestyle aspects impact the incidence of this pathology within various cultures [3,6]. Currently, ovarian cancer has not been extensively studied in the context of South American countries, with very little published literature available. The objective of this work is to provide an overview of the risk factors for ovarian cancer in South America, emphasizing those linked to social determinants, genetic components, and comorbidities. In addition, this review includes a revision of the main clinical-pathological characteristics of ovarian cancer and the risk factors most studied in other populations, contextualizing the complexity of this disease and its impact worldwide.

## 2. Methodology

An exhaustive search was conducted to identify literature to describe the ovarian cancer biology and physiopathology for new readers, in particular focused on clinical researchers. In addition, a more stringent search was performed to find studies related to the genetic components, social determinants, and comorbidities associated with ovarian cancer in South America. The search was performed through PubMed and Google Scholar, using the MeSH descriptors and keywords such as “Hereditary Breast and Ovarian Cancer Syndrome”, “Latin America”, “*BRCA1* Genes”, “*BRCA2* Genes”, and “Ovarian Neoplasms”. Additionally, the keywords “Obesity”, “chronic disease”, “Comorbidity”, “poverty”, “education”, “race”, “ethnicity”, “menarche”, “menopause”, “multiparity”, “urbanization”, “epidemiology”, and “risk factors” were utilized to capture relevant studies. Due the reported disparities in clinical trials and other biomedical studies, the Latin American population is greatly underrepresented in most of the cancer and genomic databases, including local investigations. For this reason, the number of identified reports according to the specific topics was very small for further analysis. The revision of the literature was conducted based on original articles, including cohort studies, case–control studies, cross-sectional studies, and experimental research investigating the risk factors for ovarian cancer in the South American population, and the selected articles were published within the last 10 years, with full-text availability, and were written in English or Spanish.

We considered studies involving women from 12 South American countries, without age restrictions, focusing particularly on those analyzing genetic components, socioeconomic determinants, and comorbidities. Considering the main goal of this work, we excluded studies focused on ovarian cancer treatment, research validating laboratory tests for detecting ovarian cancer-related mutations, and non-original content such as reviews, opinions, editorials, letters to the editor, and conference abstracts. Articles that did not clearly specify the region or country of interest, or those centered on education about ovarian cancer risk factors, were also excluded.

## 3. Histological and Molecular Variants of Ovarian Cancer

The World Health Organization (WHO) classifies ovarian tumors based on histological and genetic principles. This classification distinguishes between ovarian tumors originating from coelomic surface epithelial cells and non-epithelial ovarian tumors, such as those of germ cells and the sex cord–stroma. These latter tumors represent only 5% of ovarian cancers. The World Health Organization still considers this classification valid today [6].

Malignant epithelial tumors (carcinomas) are the most common ovarian cancers, accounting for 90% of cases [6,7]. There are five subtypes, and they are classified by their frequencies: serous carcinoma, which can be high-grade or low-grade (representing 70–80% and less than 5% of cases, respectively), endometrioid carcinoma (10% of cases), clear cell carcinoma (10% of cases), and mucinous carcinoma (3% of cases) [3,6]. These epithelial tumors can also be divided into two categories depending on the cause. Type I tumors are attributed to repeated cycles of ovulation, inflammation, and endometriosis, while type II tumors, diagnosed in the advanced stages, tend to be more lethal and are mainly associated with genetic mutations in the BRCA and p53 genes [3].

Serous neoplasms in the ovary present a diversity of histological characteristics. Serous cystadenomas, often unilocular cysts with smooth surfaces filled with serous fluid, can also appear as multilocular cysts; this contrasts with adenofibromas, which are predominantly solid fibrous tumors. Serous carcinomas, generally large and bilateral, show cystic, papillary, and solid growth patterns, with evident stromal invasion and the common presence of necrosis and hemorrhaging. Both serous carcinomas and adenofibromas are distinguished by their tubal-type epithelial lining, with the latter notable for the presence of psammoma bodies. The differentiation between a borderline serous tumor and serous carcinoma lies in the more evident proliferative activity and stromal invasion in the latter. These histological characteristics are crucial for the correct classification and clinical management of ovarian serous neoplasms [8].

A mucinous cystadenoma is a common ovarian neoplasm, macroscopically large and cystic. Borderline and invasive tumors have papillary and solid areas, with carcinomas being predominantly solid, with more necrosis and hemorrhaging in the invasive ones. Microscopically, mucinous tumors are characterized by glands lined by epithelial cells with intracytoplasmic mucin. Mucinous carcinomas are predominantly solid, and borderline tumors can be of the intestinal type or endocervical type (a “Müllerian mucinous borderline tumor”). The intestinal type lacks papillae or has branched papillae with atypical cells and intestinal cells [8].

Ovarian endometrioid tumors present epithelial and/or stromal elements that resemble the endometrium. Ovarian endometriosis is characterized by red, blue, and brown spots and patches, along with scars and dense fibrous adhesions; the associated cysts generally contain dark-brown fluid. Benign and borderline endometrioid tumors are rare, while an endometrioid cystadenofibroma consists of glands within a fibrous stroma [8]. Histologically, ovarian endometrioid carcinomas resemble endometrial adenocarcinomas, showing solid masses with variable consistency and cysts filled with chocolate-colored fluid. Microscopically, endometriosis reveals benign glands and stromata, while benign and borderline tumors are scarce and generally present extensive fibrous stromata. Ovarian endometrioid adenocarcinoma resembles that of the uterus, exhibiting tubular glands and often squamous differentiation in foci [8].

Clear cell tumors are characterized by clear cells containing abundant glycogen and hobnail cells, either individually or in combination [9].

Non-epithelial ovarian tumors are histologically and clinically varied, being infrequent. About 10% of patients present a non-epithelial histology, encompassing germ cell tumors and sex cord–stromal tumors. Each of these is divided into various histological subtypes and is aggressive [10]. The results of a study conducted to determine the incidence and presentation of non-epithelial ovarian tumors during 2004 and 2016 in 8917 women from different racial groups in the United States showed that 48.2% were diagnosed with sex cord–stromal ovarian cancer and 52.5% with germ cell ovarian cancer. Among the sex cord–stromal tumors, 84.1% were granulosa cell tumors, 7.9% were the Sertoli–Leydig type, 1.8% were steroid cell tumors, and 6.2% belonged to the “other” tumor category. In the case of germ cell tumors, 23.3% were classified as immature teratomas, 19.6% as dysgerminomas, 10.3% as yolk sac tumors, 8.3% as mixed germ cell tumors, 0.1% as embryonal carcinomas, and 38.3% as “other” germ cell tumor histology. In the case of germ cell tumors, 23.3% were classified as immature teratomas, 19.6% as dysgerminomas, 10.3% as yolk sac tumors, 8.3% as mixed germ cell tumors, 0.1% as embryonal carcinomas, and 38.3% as “other” germ cell tumor histology. The authors concluded that black women are more likely to be diagnosed with stromal tumors compared to white and Asian women. Additionally, black and Asian women were more likely to have germ cell cancer than white women [11].

A family history of ovarian or breast cancer emerges as the most significant risk factor for ovarian cancer. In fact, approximately 25% of all ovarian cancer cases originate from a hereditary genetic condition [12]. Most hereditary ovarian cancer cases are attributed to germline mutations in the *BRCA1* or *BRCA2* genes. Women carrying these mutations face a significantly elevated risk of developing ovarian cancer, with percentages of 50% or more for *BRCA1* and 20% for *BRCA2* [13]. Performing a salpingo-oophorectomy as a preventive measure in women with *BRCA1* or *BRCA2* gene mutations has a substantial effect by drastically reducing the risk of ovarian carcinoma and significantly lowering the overall mortality [14].

An association has been established between various genetic mutations and epithelial ovarian cancer. For high-grade serous carcinoma, mutations have been identified in the *TP53*, *BRCA1*, *BRCA2*, *NF1*, and *CDK12* genes [15]. In the case of low-grade serous carcinoma, mutations are observed in *BRAF*, *KRAS*, *NRAS*, and *ERBB2*. Endometrioid carcinoma presents mutations in the *ARID1A*, *PIK3CA*, and *PTEN* genes. Clear cell carcinoma mutations affect the *ARID1A*, *PIK3CA*, *PTEN*, *CTNNB1*, and *HNF1* genes. Finally, mucinous carcinoma exhibits mutations in the *KRAS* and *ERBB2* genes [15].

Regarding sex cord–stromal tumors, a missense point mutation (402C→G) has been detected in granulosa cell tumors in the *FOXL2* gene, which encodes essential transcription factors for the normal development of granulosa cells in the ovary [16]. Furthermore, it has been reported that 60% of Sertoli–Leydig cell tumors present somatic mutations in the *DICER1* gene [17]. This gene encodes an endoribonuclease that plays a role in the generation of mature microRNAs, which regulate gene expression through various mechanisms. Mutations in *DICER1* have the potential to be oncogenic by affecting microRNA processing [18].

## 4. Incidence and Outcome of Ovarian Cancer in the General Population

Ovarian cancer is rare in women under 40 years of age; however, in cases where it does occur in this group, most are germ cell tumors. In contrast, in women over 40 years of age, more than 90% of ovarian cancer cases are epithelial tumors, and the risk of developing them increases with age, peaking around 70 years [19]. The theory of “incessant ovulation” postulates that repeated cycles of damage caused by ovulation and the subsequent recovery of the surface epithelial layer at the ovulation site, without periods of pause induced by pregnancy, contribute to the development of ovarian cancer. Successive episodes of the apoptosis and regenerative repair of the surface epithelial cell layer at the ovulation site generate genetic instability, predisposing this cell layer to tumor formation [20].

Sex steroid hormones, including estrogens and progesterone, are of the utmost importance in the process of ovarian cancer development [20]. Estrogens have long been considered potential etiological factors in ovarian cancer [21]. Although it has been observed that the use of estrogen-containing oral contraceptives reduces the risk of ovarian cancer, this effect is mainly attributed to the reduction in the frequency of ovulation [20]. Regarding the use of hormone replacement therapy during menopause, a meta-analysis of 36 studies involving 4,229,061 participants concluded that it may increase the risk of developing ovarian cancer, particularly endometrioid and serous tumors [22].

The five-year survival rate for women with early-stage ovarian carcinoma (stage I) is over 90%, but it decreases considerably in the more advanced stages (stages III or IV), where the five-year survival rate is approximately 50%. In cases of metastatic disease, the five-year survival rate reaches only 30% [23,24]. So far, the existing early detection methods have not proven effective, resulting in late diagnoses that are associated with poor prognoses and high mortality [25].

Significant advances have been made in the treatment of ovarian cancer, with an increasing focus on personalized and targeted therapies [26]. A study, conducted over 8 years (2012–2019) and analyzing 1183 German patients with stage IV ovarian cancer according to the International Federation of Gynecology and Obstetrics staging, showed that 81.3% (962/1183) of these patients received targeted treatment during a median follow-up of 3.8 years. At the end of the follow-up, 434 (36.7%) patients were still alive, while 749 (63.3%) had died. The median overall survival was 1.9 years. It was found that age and a high-grade serous histology were determinants for survival. Patients over 80 years old had a low overall survival rate (the hazard ratio (HR) for age > 80 years vs. ≤50 years was 3.81, 95% CI: 2.76–5.27, *p* < 0.0001). Regarding the histological subtypes, survival was better for patients with high-grade serous carcinoma (*p* < 0.0001). Additionally, those who underwent surgical intervention followed by systemic treatment showed the highest survival rate, with an unadjusted HR of 0.72 (95% CI: 0.59–0.86, *p* = 0.007), compared to those who received only systemic treatment. However, after adjusting for age and histology, the differences in survival between the treatment regimens were no longer significant (*p* = 0.12) [27].

Despite its complexity, the introduction of intraperitoneal chemotherapy has represented a significant advance in improving overall survival [28]. A study conducted in Australia at a single reference center in Sydney, which evaluated the administration of intraperitoneal and intravenous platinum-based adjuvant chemotherapy in 639 patients with stage III and IV ovarian cancer over a 12-year period (2006–2018), showed a median progression-free survival of 26 months. The overall survival for the intraperitoneal group was 63.9 months, while it was 57.2 months for the intravenous group. At ten years, a significantly higher proportion of patients were still alive in the intraperitoneal group (16% vs. 3%, relative risk (RR) = 5.5, 95% CI: 1.29–24, *p* = 0.012) [29].

Although surgical cytoreduction followed by adjuvant chemotherapy remains the cornerstone of treatment, genetic testing to identify genetic mutations is now a part of standard practice [30]. In this regard, the American Society of Clinical Oncology recommends that all women diagnosed with epithelial ovarian cancer undergo germline genetic testing, especially to detect *BRCA1/2* and other genes related to ovarian cancer susceptibility. Those who do not have pathogenic or likely pathogenic *BRCA1/2* variants in their germlines should undergo somatic tumor testing to identify these variants, as these results could influence therapeutic decisions. Those with pathogenic somatic or germline variants in the *BRCA1/2* genes should receive treatments approved by the Food and Drug Administration (FDA) in both initial and recurrent settings. Additionally, for cases of clear cell, endometrioid, or mucinous ovarian cancer, somatic tumor testing for mismatch repair deficiency is recommended, as its identification offers the possibility of treatment with pembrolizumab in the context of recurrent disease. Close relatives of a patient with ovarian cancer who have a known pathogenic germline variant should receive a genetic risk assessment and personalized genetic testing. Furthermore, clinical decisions should not be based on variants of uncertain significance, and genetic testing should be performed at the time of the epithelial ovarian cancer diagnosis [12].

In Chile, in 2013, the diagnosis and comprehensive treatment of epithelial ovarian cancer in its various stages was incorporated into the Universal Access with Explicit Guarantees (AUGE-GES (Spanish acronym)) plan, providing all women with guaranteed access, opportunity, financial protection, and quality treatment [31]. Additionally, for women initially diagnosed with advanced epithelial ovarian cancer who can undergo surgery to achieve no visible tumor residue (R0), the Ministry of Health recommends opting for primary surgery rather than neoadjuvant chemotherapy (that is, chemotherapy before surgery) [32].

## 5. Social Factors and Lifestyles Associated with Malignant Ovarian Neoplasia

Ovarian cancer ranked fourth in incidence with 313,959 new cases worldwide in 2020, with an age-adjusted incidence of 6.6 per 100,000 women. In terms of mortality, it ranked third for the same year, with 207,252 deaths and an age-adjusted mortality rate of 4.2 per 100,000 women, positioning it as one of the most lethal gynecological cancers [1]. The incidence varies by country and geographic area, with the highest incidences in Eastern, Central, and Northern Europe, Polynesia, and North America, which correspond to countries with high Human Development Index levels. The lowest incidences are found in Africa and the Caribbean, countries with transitional economies [1,2].

Despite these differences in the incidence, the mortality is similar across all countries because there is no screening strategy, and the symptoms are nonspecific. Therefore, detection usually occurs at the advanced stages with a poor prognosis [3]. This situation worsens when considering the factor of unequal access to medical care, which is strongly present in lower-resource countries [2,33].

It is projected that by 2040, countries with low Human Development Index levels, defined according to the United Nations’ four Human Development Index levels (low, medium, high, very high) that include life expectancy, education, and gross national income, will experience a 96% increase in new cases and a 100% increase in deaths. This is compared to a 19% increase in new cases and a 28% increase in deaths in high-Human Development Index countries [2].

These differences related to the resources of each country could be linked to specific lifestyle-related risk factors, such as nutrition, diet, and physical activity, present in each population [2,34,35]. This is mainly because women with the same cancer characteristics, non-modifiable risk factors, and treatments may have different outcomes, suggesting that other modifiable risk factors, such as lifestyle, may influence survival [21].

Low levels of physical activity, obesity, malnutrition, breastfeeding duration, and socioeconomic status have been observed in association with a higher risk of ovarian cancer [19]. Living a healthy lifestyle incorporating health-promoting behaviors, such as physical activity, a healthy diet, and good stress management, has been associated with a lower risk of ovarian cancer [35].

Regarding food consumption, the results of a systematic review and meta-analysis of 97 cohort studies, which evaluated dietary intake and its relationship with high and low risks of ovarian cancer, showed no association between dietary intake and the risk of ovarian cancer. However, subgroup analyses indicated that the intake of leafy green vegetables (relative risk [RR] = 0.91, 95% CI: 0.85–0.98, *p* = 0.009), allium vegetables (RR = 0.79, 95% CI: 0.64–0.96, *p* = 0.021), fiber (RR = 0.89, 95% CI: 0.81–0.98, *p* = 0.014), flavonoids (RR = 0.83, 95% CI: 0.78–0.89, *p* = 0.0001), and green tea (RR = 0.61, 95% CI: 0.49–0.76, *p* = 0.0001) could significantly reduce the risk of ovarian cancer, while total fat intake (RR = 1.10, 95% CI: 1.02–1.18, *p* = 0.009), saturated fat (RR = 1.11, 95% CI: 1.01–1.22, *p* = 0.023), saturated fatty acids (RR = 1.19, 95% CI: 1.04–1.36, *p* = 0.010), cholesterol (RR = 1.13, 95% CI: 1.04–1.22, *p* = 0.0022), and retinol intake (RR = 1.14, 95% CI: 1.00–1.30, *p* = 0.048) could significantly increase the risk of ovarian cancer [36].

Another more recent systematic review and meta-analysis, which evaluated dietary fats and key serum lipids that increase the risk of ovarian cancer from 18 cohort studies and 23 case–control studies (109,507 ovarian cancer patients and 2,558,182 control patients without ovarian cancer), showed that individuals with higher dietary intakes of total fat (RR = 1.19, 95% CI: 1.06–1.33), cholesterol (RR = 1.14, 95% CI: 1.03–1.26, I^2^ = 19.4%), saturated fats (RR = 1.13, 95% CI: 1.04–1.22, I^2^ = 13.4%), and animal fat (RR = 1.21, 95% CI: 1.01–1.43, I^2^ = 70.5%) have a significantly higher risk of developing ovarian cancer. Additionally, higher serum triglyceride levels (RR = 1.33, 95% CI: 1.02–1.72, I^2^ = 89.3%) present a higher risk of ovarian cancer [37].

A sedentary lifestyle represents another risk factor for the development of ovarian cancer. A systematic review and meta-analysis that evaluated seven studies and included 2060 cases of ovarian cancer (three prospective cohort studies and four case–control studies) showed a statistically significant association between high versus low levels of sedentarism, increasing the risk of ovarian cancer by 29% (RR = 1.29, 95% CI: 1.07–1.57) [38]. Another study, which reviewed 34 articles and meta-analyses to investigate the association between physical activity, physical inactivity, and the risk of ovarian cancer, revealed that moderate and low physical activity (2 to <4 h/week) helps reduce the risk of ovarian cancer by 31% (RR = 0.69, 95% CI: 0.56–0.85, *p* < 0.05), and moderate physical inactivity (≥3 and <7 h/day) increases the risk of ovarian cancer (RR = 1.14, 95% CI: 1.06–1.23, *p* < 0.05) [39]. The associations between healthy dietary components before diagnosis and ovarian cancer survival raise the possibility that dietary choices after diagnosis may improve survival. Identifying modifiable lifestyle factors that improve ovarian cancer survival is crucial.

Sociodemographic factors, such as education and geographic region, also influence the likelihood of a woman developing ovarian cancer [40]. A retrospective case–control study, conducted at the Oncology Referral Hospital in West Java, Indonesia, which included 408 women, found that the variables significantly associated with an increased risk of developing ovarian cancer were advanced age (≥45 years), low education, obesity, previous surgery, parity, and poor sleep quality [41]. Another study in the United States analyzed the combined effect of the sociodemographic characteristics of women’s areas of residence on their overall survival. It revealed significant disparities in the survival rates between counties classified as disadvantaged and those classified as affluent based on social and economic factors, education, and access to care. Women living in the most economically and socially deprived counties were found to have a 25% higher mortality risk of ovarian cancer than patients living in counties with the highest socioeconomic status and best access to care [42].

Concerning obesity, one study explored the association between the ovarian cancer survival, stage at diagnosis, histotype, and reproductive, anthropometric, and lifestyle factors in a cohort of over 1 million women in the United Kingdom who completed a health questionnaire between 1996 and 2001 and had an average follow-up of 17.7 years. The results showed that 13,222 women (1.1%) were diagnosed with ovarian cancer, of which 8697 (66%) died from the disease. The stage at diagnosis was an important determinant of survival (stage IV versus I, RR = 10.54, 95% CI: 9.16–12.13). Survival by specific histotype was worse for high-grade tumors than for low-grade ones. Survival appeared to worsen the older the age at diagnosis (per 5 years, RR = 1.19, 95% CI: 1.15–1.22) and with a high pre-diagnosis BMI with an overall 6% higher risk for each five-unit increase in BMI, and it was only statistically significant for serous carcinomas (per five-unit increase in BMI, RR = 1.06, 95% CI: 1.02–1.11). Smoking also showed worse survival (current versus never: RR = 1.17, 95% CI: 1.07–1.27). No evidence was found of an association between reproductive, anthropometric, and other lifestyle factors (alcohol, physical exercise, education level) before diagnosis [4].

Another study involving 461,646 women (≤49 years of age) enrolled in the Danish Medical Birth Registry found that the risk of premenopausal ovarian cancer increased by 23% for each 5 kg/m^2^ increase in BMI. Compared to normal weight, obesity was associated with high rates of premenopausal ovarian cancer [43]. A systematic review and meta-analysis of overweight and obese women that included 15 cohorts and 26 case–control studies with 28,741 cases of ovarian cancer showed relative risks of ovarian cancer for overweight and obesity cases of 1.06 (95% CI: 1.00–1.12) and 1.19 (95% CI: 1.11–1.28), respectively. Among premenopausal women, a higher risk of ovarian cancer was observed in overweight women (RR = 1.34; 95% CI: 1.03–1.75) and obese women (RR = 1.51; 95% CI: 1.21–1.88). For postmenopausal women, there was no statistically significant association between being overweight (RR = 1.00; 95% CI: 0.87–1.14) and obese (RR = 1.03; 95% CI: 0.82–1.31) [44]. The underlying biological mechanisms of this relationship include insulin resistance, hyperinsulinemia, elevated levels of growth factors, chronic inflammation, and alterations in sex hormone levels [45]. Therefore, addressing obesity becomes crucial for both prevention and improving outcomes in cases of ovarian cancer.

It is interesting to consider that socioeconomic status is one of the predictors of the incidence and survival of ovarian cancer, mainly due to its relationship with timely access to healthcare, patient awareness of ovarian cancer symptoms, a timely response to symptoms, and lifestyle, among others. This may also be greatly affected by the geographic region in which the woman lives.

## 6. Genetic Components of Ovarian Cancer in South America

South America is below the global incidence rate (6.6/100,000 women), with an age-standardized incidence of 5.8 per 100,000 women and a mortality rate of 3.6 per 100,000 women. Similarly, Chile has an age-adjusted incidence and mortality rate similar to the South American rates of 6 per 100,000 women and 3.6 per 100,000 women, respectively. Still, ovarian cancer in the region ranks fourth in incidence nationally among gynecological cancers and third in terms of mortality [46].

A 2018 study in Colombia, aiming to describe the frequency and type of pathogenic germline mutations in breast and ovarian cancer susceptibility genes, showed that nineteen patients (22.4%) carried a harmful germline mutation in a cancer susceptibility gene: *BRCA1*, *BRCA2*, *PALB2*, *ATM*, *MSH2*, or *PMS2*. The research demonstrated that the frequency of *BRCA1/2* mutations was 17.6% in ovarian and breast cancer [47]. Another study detected three variants of uncertain significance in *BRCA1*, and, after in silico analysis, it was concluded that c.8112C>G and c.3119G>A (p.Ser1040Asn) are probably harmful, while c.3083G>A (p.Arg1028His) is probably neutral. Regarding *BRCA2*, three variants of uncertain significance were identified, and the in silico analysis suggested that c.865A>G (p.Asn289Asp) and c.6427T>C (p.Ser2143Pro) are probably harmful, while c.125A>G (p.Tyr42Cys) is probably neutral. Additionally, 13 polymorphisms (4 in *BRCA1* and 9 in *BRCA2*) were identified, of which 2 are associated with a moderate increase in breast cancer risk (*BRCA2* c.1114A>C and c.875566T>C) [48]. Furthermore, a Latin American study included 78 cases from Colombia. The results showed a 13% prevalence of pathogenic variants associated with the *BRCA1* and *BRCA2* genes [49]. Another Latin American study enrolled 79 patients from Colombia diagnosed with high-grade serous ovarian cancer, with a mean age of 57.8 years at diagnosis. The results revealed a 23% prevalence (23 out of 79) of pathogenic variants associated with the *BRCA* gene [50].

Similarly, three studies conducted in Argentina have demonstrated the persistence of germline mutations in *BRCA1/2* in the region. One study conducted in Argentina that included 155 women with breast cancer, ovarian cancer, and both, with an average age of 42 years (range: 28–81 years), showed a prevalence of 25.8% of *BRCA1* and *BRCA2* mutations among patients who met the criteria for hereditary ovarian cancer [51]. Another Latin American study included 57 cases from Argentina. The results showed a 25% prevalence of pathogenic variants associated with the *BRCA1* and *BRCA2* genes [49]. Similarly, a separate study enrolled 110 patients diagnosed with high-grade serous ovarian cancer, with a mean age of 57.8 years at diagnosis. The results revealed a 31% prevalence (34 out of 110) of pathogenic variants associated with the *BRCA* gene [50].

Regarding other Hispanic countries, in Mexico, BRCA mutations were identified in a Latin American study that enrolled 222 patients with Hereditary Breast and Ovarian Cancer, 68 of whom were from Mexico. The results showed an 18% prevalence (12/68) of the pathogenic variant [49]. Another study included 48 patients from Mexico, 116 patients from Brazil, and 31 patients from Peru, all diagnosed with high-grade serous ovarian cancer. The results revealed a 40% prevalence of pathogenic variants associated with the BRCA gene in the Mexican group (19/48), a 245 prevalence in the Brazilian group (28/116), and a 42% prevalence in the Peruvian group (13/31) [50]. A more recent study on the characterization of BRCA variants included 382 patients with ovarian cancer. The results showed a 20.7% prevalence of pathogenic variants in *BRCA1/2*. The most frequent pathogenic variants detected in *BRCA1* were c.2105dupT, c.68_69delAG, c.140G>T, and c.815_824dupAGCCATGTGG, while in *BRCA2* they were c.8023A>G, c.6024dupG, and c.9235delG [52].

Studies on the current Chilean population performed using classical genetic markers have established that the Chilean population originated primarily from the admixture of European people, particularly Spaniards and Amerindians [53]. A study on the retrospective analysis of the registry of the High-Risk Breast and Ovarian Cancer Program of a single private clinical center in Chile (years 2008–2018) demonstrated that most variants (81.8%) in 315 women were in *BRCA1* or *BRCA2*, and that 10.5% of cases were carriers of a pathogenic or probably pathogenic variant in genes other than *BRCA1/2*, such as *RAD51C*, *RAD51D*, *ATM*, *PALB2*, *CHEK2*, and *CDH1* [54]. A retrospective study analyzed genetic test results to identify the genotypic spectrum of *BRCA1/2* variants in Chilean families between 2016 and 2021 from a single healthcare center. During the analyzed period, 891 tests were indicated, with 722 conducted primarily on women (91.1%) and patients diagnosed with cancer (75.2%). Among those diagnosed with cancer, 96% were female, with 92.2% had breast and/or ovarian cancer. Of the 501 cases of breast and/or ovarian cancer, 20.5% presented pathogenic variants associated with the *BRCA* gene (103/501) [55].

In general terms, the incidence of germline mutations or other risk factors for hereditary ovarian cancer does not exceed 10% in the Latin population, suggesting that additional factors still promote this neoplasm within the region.

Extrinsic and intrinsic host factors have been observed in several populations around the world. Table 1 displays a summary of the most relevant findings related to the ovarian cancer risk in South American countries, excluding French Guyana. Unfortunately, very few articles have been published in the last five years, and their scatter content may not represent all aspects of this cancer type.

## 7. Social Determinants and Ovarian Cancer and Other Neoplasms in South America

In Colombia, a descriptive, cross-sectional, and ecological study that included 36,798 women diagnosed with ovarian cancer from the regions of Antioquia, Santander, and Bogota between 2009 and 2016 revealed that the predominant sociodemographic and economic factors in the studied population included a high percentage of married women, low educational levels, and limited access to health services [61].

In Ecuador, a study involving 84 patients with an average age of 56.5 years (95% CI: 53.7–59.3) revealed that multiparity, despite being considered a protective factor against ovarian cancer, did not behave as expected, as 60.7% of multiparous women had ovarian cancer. Similarly, late menopause (>52 years) was only present in 8.3% of women with ovarian cancer. However, regarding age, they found that the incidence of ovarian cancer increases after 50 years, which agrees with the existing evidence [63]. In another study that estimated the 2- and 4-year survival in 174 patients with ovarian cancer who underwent primary cytoreduction between 2016 and 2018, it was described that 58.6% of women with ovarian cancer lived in an urban geographical area, 62.63% had no comorbidities, and 27% of cases had high-grade serous carcinoma with stage III (33.9%). Additionally, 60.9% of patients received complete chemotherapy and 55.2% underwent suboptimal cytoreductive surgery. The results showed that the overall survival of the women was 73% at 2 years and 44% at 4 years [63,64]. 

In Brazil, an exploratory study aimed at analyzing trends in ovarian cancer mortality and its relationship with age, education, and race, with data collected from the Brazilian mortality information system between 2006 and 2016, showed that ovarian cancer was responsible for 34,003 deaths during the period, and that the mortality rate per 100,000 women varied depending on age (0.46 in women under 40 years; 4.2 between 40 and 59 years; 12.2 between 60 and 79 years; and 19.4 in women aged 80 years or older). Race was declared in 94.5% of the population, and among it, 65% of women with ovarian cancer were white; only 35% of deaths occurred among non-white women, classified as black, Asian, mixed-race, or indigenous. Education level data were found in 25% of the population and, of this population, only 26% had 8 years or more of schooling. Therefore, women with limited education are more susceptible to a worse ovarian cancer diagnosis [58]. 

**Figure 1:**
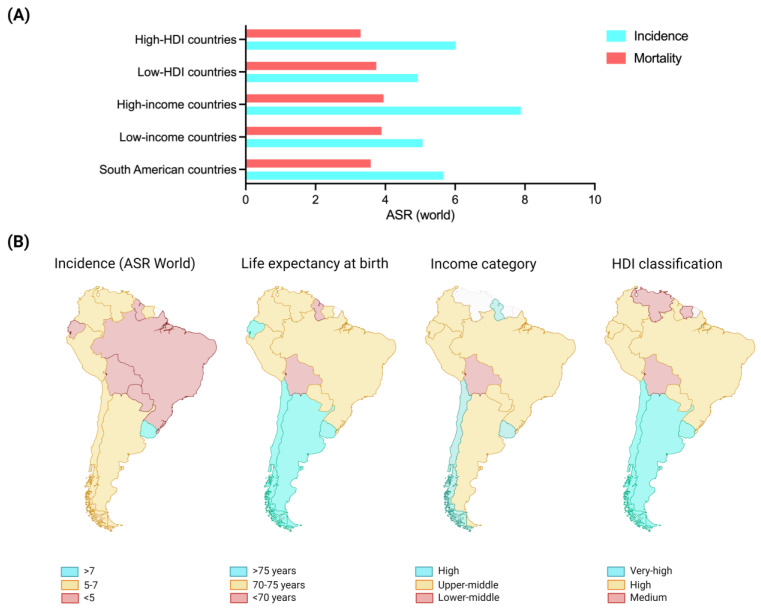
(**A**) Ovarian cancer incidence rates in South America, according to income and human development classification. The bars represent the reported mean value of ovarian cancer incidence in women from South America and the measured incidence in other countries grouped by their income or HDI-classification. (**B**) The map shows the distribution of ovarian cancer cases in South America and its graphical correlation with life expectancy and economic status. Data source: World Health Organization—Global Health Observatory (2024), and HDI source: United Nations Development Programme (2022). ASR: age-standardized rate (world) for ovarian cancer. HDI: Human Development Index. Countries without information were removed from the map.

Figure 1 represents the age-standardized incidence and mortality rates for ovarian cancer for the South American region and its comparison with those observed for the whole group of countries classified by their levels of income and human development. While Figure 1A presents this summarized information, Figure 1B displays same parameters in a more visual manner for each country within South America, including their reported life expectancies (Figure 1B). These contrasts must be considered to understand the lack of literature within the region.

## 8. Comorbidities in South America Associated with Ovarian Cancer

Multiple studies have evaluated the relationship between metabolic diseases, obesity, and lifestyle with different types of cancer in the Latin American population, but very little has been considered in terms of ovarian neoplasms.

A retrospective design study covering the period 2012–2019 with 112 cancer patients in Argentina, evaluating factors such as being overweight and obese and presenting metabolic syndrome, showed that the most frequent neoplasms associated with being overweight were breast cancer, digestive system cancer, ovarian cancer, prostate cancer, and thyroid cancer [56].

However, a cross-sectional study conducted in Ecuador during the period from January 2013 to December 2017, which examined 29 virgin women with ovarian cancer, revealed that the most prevalent chronic diseases were diabetes mellitus and hypertension, and the most significant associated risk factor was overweight and obesity [62].

At the South American level, the most relevant data in terms of the number of cases have been developed in Brazil. A study indicated that 3.8% of all cancer cases in 2012 were attributed to a high BMI, with a greater burden in women (10,059 cases, equivalent to 5.2%) compared to men (5406 cases, equivalent to 2.6%). Additionally, it was observed that a BMI above 22 is related to ovarian cancer in women over 50 years old [57].

In Chile, the high prevalence of overweight and obesity affects three out of four people, making it one of the highest rates on the continent. This problem reflects inequalities in distribution, driven by structural determinants such as social security, socioeconomic levels, education, and gender. Although Chile is classified as a high-income economy, the country’s high socioeconomic inequality presents an additional challenge for health interventions [65]. According to the most recent National Health Survey (2017), 74.2% of the population over 15 years old in Chile had high body mass indexes (39.8% overweight and 34.4% obese), significantly exceeding the global average for adults in the same period, which was 38.9% [66]. Recently, a study that examined electronic records of patients with confirmed epithelial ovarian cancer who received care between 2004 and 2017 revealed that body composition, especially the presence of high visceral adiposity estimated by computed tomography, is associated with worse survival in cases of epithelial ovarian cancer [60].

The relationship between risk factors related to metabolic and cardiovascular disease with ovarian cancer incidence and mortality rates requires more studies and larger number of participants. We have summarized the data available from open sources (Table 2) regarding classic cancer risk factors within the twelve South American countries. It seems that the highest ovarian cancer incidence rates may correlate with the HDI-value than any other parameter.

## 9. Conclusions

Based on the revised articles, it can be concluded that although there are reports on the prevalence of mutations in the *BRCA1* and *BRCA2* genes in South American countries, such as Chile, Colombia, Argentina, Uruguay, and Brazil, including Mexico as Hispanic, the general prevalence is close to 15–20% in women with hereditary risk factors or those diagnosed with ovarian and/or breast cancer. However, it is important to highlight that the available studies are limited. More research is needed to accurately determine the prevalence of the punctual mutation in each country, as well as those mutational signatures that could be unique for the Latin American population.

In terms of the social, demographic, and economic factors that influence the incidence of ovarian cancer, these vary by country, but similarities are observed in age, being more prevalent in women over 50 years old, and in women with poor education and difficulties in accessing healthcare.

Similarly, the absence of studies regarding the effect of contraception and hormone replacement therapy in the Latin American population is highlighted, despite being widely studied factors in the international scientific literature. This gap in the research underscores the need to address these aspects in future research in the region.

This article has several limitations, starting with the very limited number of studies published and the number of participants in each study. The revised literature covers several years of research conducted in each country; these years may represent disparities in funding for biomedical research, the number and capabilities of each research group and the possibility of enrolling patients, and the access to technological facilities. For now, it is not possible to conduct a more comprehensive analysis; however, this work may promote the interest of other researchers to collaborate in the systematic revision of current and future data.

When socioeconomic aspects, such as the human development or income per country, are used to compare the incidence of ovarian cancer and the mortality of diagnosed women in South America, extreme patterns are not observed (Figure 1). However, when these social determinants are broken down for each country within South America, substantial differences are revealed that may explain our observations from the available literature.

Regarding the need to improve lifestyles as a preventive measure to reduce the possibility of morbidities and the concomitant development of ovarian cancer, although the literature may not be sufficient to conclusively establish a direct link between obesity and/or diabetes and the development of ovarian cancer, the indirect association suggests the importance of conducting additional clinical studies. These studies could confirm or dismiss the hypothesis that obesity and diabetes could be related to the risk of developing ovarian cancer, thereby providing a more solid basis for future interventions and prevention strategies.

## Figures and Tables

**Table 1:** Overview of reported risk factors for ovarian cancer in South American women.

Country		Studied Factors	
	Genetic Background *	Health Status at Diagnosis	Social and Demographic Factors
Argentina	25.8% (155 cases) [51] 25% (57 cases) [49] 31% (110 cases) [50]	Elevated weight (112 pooled patients, 63.4% women, different tumor types) [56]	Women in their fifth decade (155 pooled cases, 27 ovarian) [51]
Brazil	24% (116 cases) [50]	BMI > 22 in women > 50 years old (4682 ovarian cancer cases) [57]	Low educational level; white/Caucasian ancestry (34,003 ovarian cancer cases) [58] Mortality related to asbestos (58,182 ovarian cancer deaths) [59]
Chile	10.5% (315 cases) [54] 20.5% (501 cases) [55]	High visceral adiposity and poor survival (538 ovarian cancer cases) [60]	No data
Colombia	17.6% (85 cases) [47] 6.9% and 13.8% *BRCA1* and *BRCA2* (72 cases) [48] 13% (78 cases) [49] 23% (79 cases) [50]	No data	Married women, low educational level, limited healthcare access (36,798 ovarian cancer cases) [61]
Ecuador	No data	Hypertension and diabetes (394 women, 124 ovarian) [62]	Age (>50 years) and multiparity. (84 women) [63] Age (>50 years), urban area. (174 cancer patients) [64]
Peru	42% (31 cases) [50] 20.7% (382 cases) [52]	No data	No data

* Observed prevalence, percentage of mutated *BRCA1/2* genes in combined groups (ovarian and breast cancer). BMI: body mass index. All gaps were intentionally left to reinforce the absence of studies on these areas. References are indicated with brackets.

**Table 2:** Summary of the incidence and mortality of ovarian cancer and key biological–social characteristics of women from South American countries.

Country	Incidence	Mortality	Overweight	Obese	Smoking	Exercise	Hypertension	HDI Value
Argentina	6.8	4.1	65.1	36.3	17.3	40.1	41.2	0.849
Bolivia	3.1	2.2	68.7	34.3	3.8	26.6	27.2	0.698
Brazil	5.1	3.3	64.4	31.7	7.8	45.1	42.1	0.760
Chile	6.3	3.6	79.0	44.3	24.4	45.9	33.1	0.860
Colombia	6.7	4.1	62.9	28.9	3.8	40.5	30.8	0.758
Ecuador	5.1	3.2	69.4	32.3	2.2	27.8	25.1	0.765
Guyana	4.5	3.5	66.5	38.2	1.9	45.1	41.8	0.742
Paraguay	4.8	3.0	71.3	35.9	3.2	39.8	50.9	0.731
Peru	5.8	3.6	70.6	31.5	2.3	36.9	18.4	0.762
Suriname	5.5	3.9	67.2	38.5	ND	55.1	43.3	0.690
Uruguay	8.0	4.6	65.0	35.6	16.1	36.8	38.9	0.830
Venezuela	6.1	3.8	53.8	24.9	ND	52.8	39.1	0.699

Incidence and mortality: 2022 report, age-standardized rate (ASR world) per 100,000. Mortality, females, and ovarian cancer; overweight: 2022 report, prevalence of overweight among female adults, BMI ≥ 25 (age-standardized estimate) (%); obesity: prevalence in 2022 of obesity among female adults, BMI ≥ 30 (age-standardized estimate) (%). BMI: body mass index; exercise: 2022 report, prevalence of insufficient physical activity among female adults aged 18+ years (age-standardized estimate) (%); smoking: by 2022, estimate of current cigarette smoking prevalence in women (%) (age-standardized rate); hypertension: 2019 report, prevalence of hypertension among women aged 30–79 years, age-standardized. HDI: Human Development Index. Data source: cancer TODAY − IARC/data version Globocan 2022 (v1.1—8 February 2024); World Health Organization—Global Health Observatory (2024) (https://www.who.int/data/gho/data/indicators. Accessed on: 29 June 2024). HDI Source: United Nations Development Programme (2022).

## Data Availability

No new data were created or analyzed in this study. Data sharing is not applicable to this article.
